# The Profound Impact of the COVID-19 Pandemic on the Epidemiology of Quadriceps and Patellar Tendon Ruptures: Insights From a Single Trust in the United Kingdom

**DOI:** 10.7759/cureus.98022

**Published:** 2025-11-28

**Authors:** Thivagar Murugesan, Hidayatul Rasyidah Syida Abdullmalek, Suresh Kondi, Hamood Ur Rehman, Mike Carmont, Catriona Heaver, Tosan Okoro

**Affiliations:** 1 Orthopaedic Surgery, Shrewsbury and Telford Hospital NHS Trust, Shrewsbury, GBR; 2 Trauma and Orthopaedics, Shrewsbury and Telford Hospital NHS Trust, Shrewsbury, GBR; 3 Foot and Ankle Surgery, The Robert Jones and Agnes Hunt Orthopaedic Hospital, Oswestry, GBR; 4 Orthopaedics, The Robert Jones and Agnes Hunt Orthopaedic Hospital, Oswestry, GBR

**Keywords:** covid-19, epidemiology, patellar tendon rupture, physical inactivity, quadriceps tendon rupture, rehabilitation, tendon injury

## Abstract

Introduction

Quadriceps and patellar tendon ruptures are uncommon but disabling injuries that require surgical repair. Changes in physical activity patterns during and after the COVID-19 pandemic may have influenced their occurrence. This study compares the incidence, demographics, and seasonal distribution of these injuries before and after the pandemic within a single UK NHS trust.

Methods

A retrospective review was performed of all patients undergoing primary repair of quadriceps or patellar tendon ruptures at the Shrewsbury and Telford NHS Trust from January 2014 to December 2024. The pre-COVID period (2014-2019) was compared with the post-COVID period (2021-2024), with 2020 excluded due to major service disruption and atypical clinical pathways during the first pandemic year. Annual incidence rates were calculated as cases per 100,000 population, using year-specific catchment population estimates from the Office for National Statistics (ONS). Demographic variables, injury characteristics, and seasonal patterns were extracted from electronic records. Between-period comparisons used the Mann-Whitney U test. No adjustment for age, BMI, or comorbidities was performed.

Results

A total of 203 patients were identified, including 95 pre-COVID and 108 post-COVID. The median age was 63 years (range 15-90), with a male-to-female ratio of 7.8:1. Injuries occurred most frequently in autumn (28%). The median annual incidence increased from 4.6 per 100,000 pre-COVID to 8.3 per 100,000 post-COVID (incidence rate ratio 1.8, p = 0.0073). The median number of cases per year rose from 16 to 27 between periods.

Conclusion

The incidence of surgically treated quadriceps and patellar tendon ruptures increased in the post-COVID period. Although the study does not adjust for potential confounders, the findings suggest an association between the post-pandemic era and higher injury rates. Further research with multivariable analysis is needed to clarify contributing factors.

## Introduction

Quadriceps and patellar tendon ruptures are uncommon but serious injuries that disrupt the knee extensor mechanism and typically require surgical repair [1]. Quadriceps tendon ruptures occur more often in adults over 40 years of age, whereas patellar tendon ruptures are more frequently seen in younger, active individuals [2,3]. Male sex and comorbidities such as diabetes, chronic kidney disease, obesity, and corticosteroid use are recognised risk factors that contribute to tendon weakening and predispose individuals to rupture [4-6].

The COVID-19 pandemic introduced marked changes in physical activity patterns. Periods of reduced activity due to lockdowns, along with abrupt or unsupervised increases in exercise among some individuals, may have contributed to deconditioning or altered loading patterns that affect tendon health [7-9]. Reports of rising rates of other tendon injuries, such as Achilles and rotator cuff ruptures, have raised the possibility of similar trends in lower-extremity extensor mechanism injuries, although evidence specific to quadriceps and patellar tendon ruptures remains limited [10]. In addition, pandemic-related healthcare disruptions may have influenced presentation patterns and access to care [11].

Seasonal variation has also been described in tendon injuries, with higher rates reported during colder months, though it is unclear whether these patterns have changed in recent years [12].

Given these uncertainties, further evaluation of injury trends across the pre- and post-pandemic periods is warranted. This study aims to compare the incidence, demographic characteristics (age, sex), and seasonal distribution of surgically treated quadriceps and patellar tendon ruptures between the pre-COVID (2014-2019) and post-COVID (2021-2024) periods.

## Materials and methods

Study population and setting

All patients who underwent primary surgical repair of quadriceps or patellar tendon ruptures between January 2014 and December 2024 at the Shrewsbury and Telford NHS Trust were identified retrospectively using the Bluespier electronic database. The Trust comprises two district general hospitals serving the Shropshire region, which had an estimated mid-2023 population of 329,260 based on Office for National Statistics (ONS) mid-year estimates [13]. For analysis, 2014-2019 was defined as the pre-COVID period and 2021-2024 as the post-COVID period.

Exclusion criteria

Patients were excluded if they (1) did not undergo primary repair despite having a documented quadriceps or patellar tendon rupture, (2) presented outside the study periods or within 2020 (to minimise confounding from pandemic-related service disruptions), (3) had incomplete documentation preventing confirmation of diagnosis or extraction of study variables, or (4) sustained tendon injuries due to high-energy trauma, polytrauma, or penetrating mechanisms, as these represent different injury patterns compared with typical degenerative or low-energy ruptures.

Diagnosis

Diagnosis was recorded from clinical documentation. Most cases were diagnosed clinically, with imaging (plain radiographs and, when required, ultrasound or MRI) used selectively to support clinical assessment rather than to define diagnostic pathways.

Data extraction

Electronic medical records were reviewed to extract demographic variables (age, sex), injury characteristics (laterality and seasonal timing), and operative details. All data were anonymised prior to analysis.

Annual incidence rates were calculated as the number of surgically treated quadriceps or patellar tendon ruptures per 100,000 population, using year-specific ONS mid-year estimates for the Shropshire region as the denominator.

Statistical analysis

Descriptive statistics and graphical outputs were generated using Microsoft Excel, and comparative analyses were performed using SPSS (IBM Corp., Armonk, NY). Crosstabulations and Mann-Whitney U tests were used where appropriate, with statistical significance set at p < 0.05.

Ethical approval

This study involved a retrospective review of anonymised records and required no formal ethics or institutional review board approval under local regulations.

## Results

**Figure 1:**
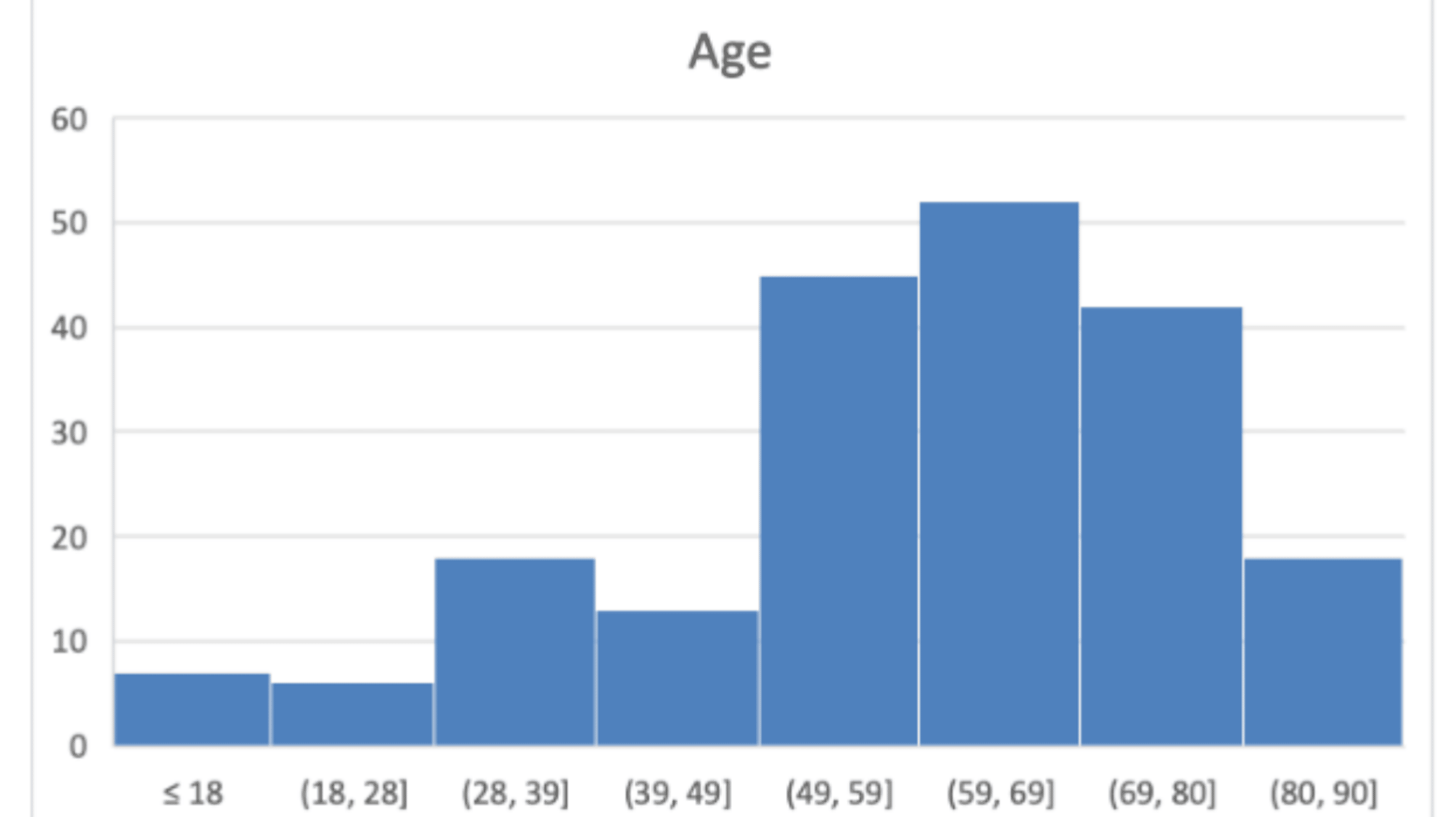
Age distribution of all patients who sustained knee extensor mechanism injuries. X-axis: Age of patients included in the study; Y-axis: Number of patients with knee extensor mechanism injuries.

A total of 203 patients underwent primary repair of quadriceps or patellar tendon ruptures between 2014 and 2024, with 95 cases occurring in the pre-COVID period and 108 cases in the post-COVID period. The median age was 63 years (range 15-90), and the male-to-female ratio was 7.8:1, a pattern consistent across both time periods. Figure 1 shows the age distribution of all patients included in the study.

**Figure 2:**
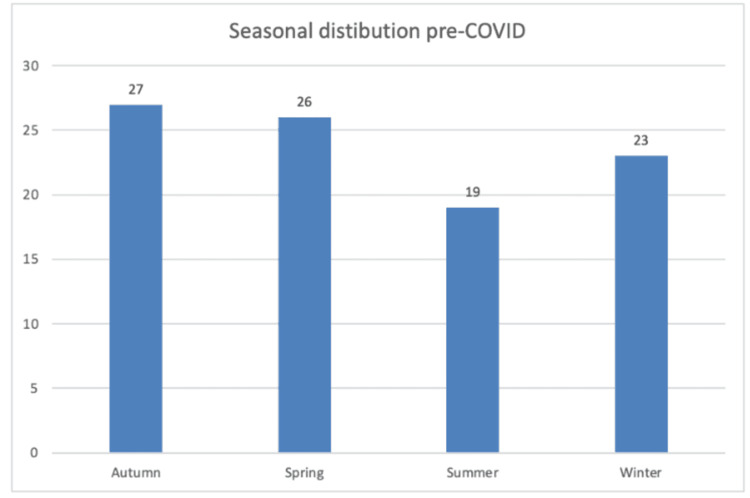
Seasonal distribution of injuries in the pre-COVID pandemic period (2014-2019), showing the highest proportion of injuries occurring in autumn. X-axis: Seasons; Y-axis: Number of patients with knee extensor mechanism injuries.

**Figure 3:**
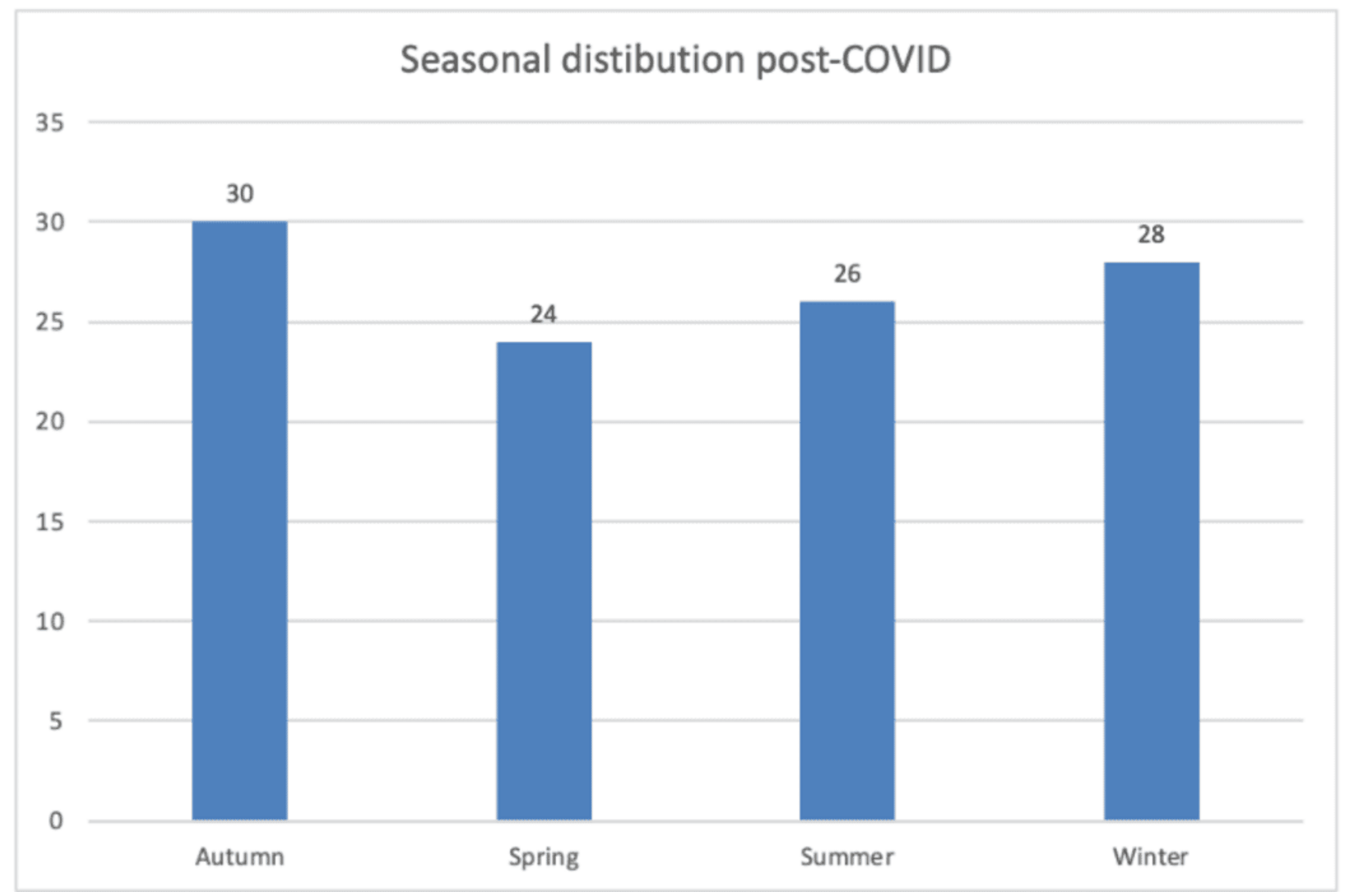
Seasonal distribution of injuries in the post-COVID pandemic period (2021-2024), with the highest incidence recorded in autumn (29.41%). X-axis: Seasons; Y-axis: Number of patients with knee extensor mechanism injuries.

Seasonal analysis demonstrated that autumn accounted for 28% of all cases, followed by winter (25.1%), spring (24.6%), and summer (22.2%). This distribution was similar in both periods, with autumn remaining the season with the highest proportion of injuries. Figures 2-3 illustrate the seasonal distribution for the pre- and post-COVID periods, respectively.

**Figure 4:**
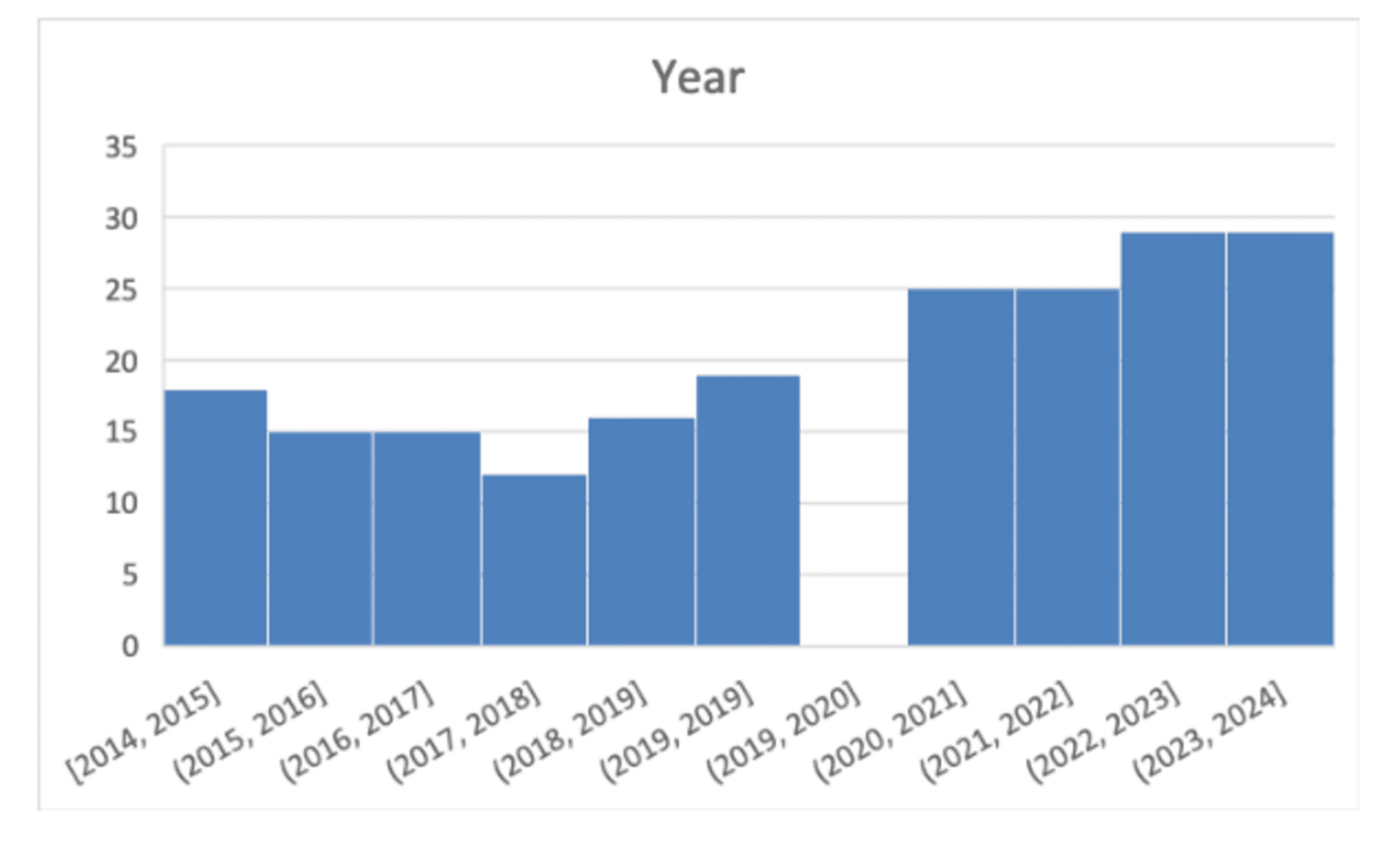
Incidence of knee extensor mechanism injuries per year. X-axis: Years; Y-axis: Number of patients with knee extensor mechanism injuries.

The median annual incidence increased from 4.6 per 100,000 in the pre-COVID period to 8.3 per 100,000 in the post-COVID period, corresponding to an incidence rate ratio of 1.8. This difference was statistically significant (Mann-Whitney U test, p = 0.0073). Annual incidence values are shown in Figure 4.

The median number of cases per year rose from 16 per year in the pre-COVID period to 27 per year in the post-COVID period.

## Discussion

This study demonstrates an increase in surgically treated quadriceps and patellar tendon ruptures in the post-COVID period compared with the pre-COVID years. Similar trends have been reported for other tendon injuries. For example, Carmont MR et al. observed a rise in acute Achilles tendon ruptures in the UK after the pandemic, and Bi AS et al. noted an increase in primary repair procedures for Achilles ruptures in the United States [14,15]. Evidence specifically examining quadriceps and patellar tendon ruptures remains limited, as most publications group these injuries with other tendon pathologies and report data only up to 2021 [16,17]. The continued increase observed in our cohort through 2024 has not previously been described.

In our region, the peak incidence in 2022 aligns temporally with the phased lifting of national restrictions in the UK, which concluded by early 2022 [18]. Although causation cannot be established, this timing mirrors patterns suggested in other musculoskeletal studies, where periods of reduced activity followed by a sudden resumption of normal routines have been associated with higher injury rates. Shropshire’s comparatively older population may also contribute to the age distribution seen in our cohort.

Pandemic-related lifestyle changes likely played a contextual role, although they cannot be directly assessed with the available data. Several studies have documented reduced physical activity during lockdown periods [19,20]. Increased sedentary behaviour, including prolonged sitting time and greater screen use, has also been widely reported [21,22]. Evidence further suggests a decline in overall cardiovascular fitness and participation in routine exercise during this time [7,8]. Weight gain was observed across many populations throughout the pandemic [23,24], and such changes may contribute to increased mechanical load on tendons [25]. These factors collectively provide plausible contextual background for post-pandemic increases in tendon injury, but they were not measured in this study and should therefore be interpreted cautiously.

The seasonal trend identified, an increased proportion of ruptures occurring in autumn, has also been described in other tendon injury research. Cooler temperatures may reduce tissue elasticity, and lower levels of warm-up or outdoor activity during these months may contribute to injury risk. These explanations were not examined directly in our dataset but may help contextualise the consistent autumn predominance observed before and after the pandemic.

Several limitations must be acknowledged. First, only operatively managed cases were included, which may underestimate true incidence, although operative repair is standard for quadriceps and patellar tendon ruptures and most cases are likely to be captured. Second, the pre- and post-COVID comparison periods were of unequal duration, which may introduce variability despite the use of annualised incidence rates. Third, we were unable to adjust for comorbidities, BMI, activity levels, or detailed injury mechanisms, limiting the ability to evaluate contributing factors or assess confounding. These limitations restrict causal interpretation and highlight the need for prospective studies using multivariable methods.

## Conclusions

A significant increase was noted in the incidence of quadriceps and patellar tendon rupture rates following the COVID-19 pandemic. This study provides detailed epidemiological data on age, sex, and seasonal distribution, filling a gap in the existing literature and laying the groundwork for future research into the impact of population-level lifestyle changes on tendon health. The findings highlight the importance of anticipating tendon injuries in older, predominantly male patients, particularly during periods of abrupt lifestyle changes. Clinicians should consider strategies for gradual reconditioning and a safe return to activity to reduce the risk of tendon rupture following prolonged inactivity.
